# Improving sustainability in sexual health: A pilot project reintroducing reusable stainless steel vaginal specula at a sexual health clinic

**DOI:** 10.1177/09564624241298873

**Published:** 2024-11-05

**Authors:** Isobel Hall, Gillian Dean, Amanda Clarke

**Affiliations:** 1 12190 Brighton and Sussex Medical School, Brighton, UK; 2 Brighton and Hove Sexual Health and Contraception Service, University Hospitals Sussex, Brighton, UK

**Keywords:** Europe < location, environment < other, supplies < other, equipment < other

## Abstract

**Introduction:**

In line with the NHS net zero initiative, University Hospitals Sussex (UHS) committed to a Green Plan which included the introduction of reusable instruments. Following positive responses from a staff and patient survey in 2021, the sexual health and contraception (SHAC) department began a pilot scheme of 100 medium size reusable stainless steel vaginal specula (RMS) as an alternative to disposable acrylic models. The aim was to determine outcomes regarding sustainability goals and clinician experiences.

**Methods:**

Estimated carbon footprint and cost was calculated for actual use during study period, and expected use if RMS were to be exclusively used in the future. A staff questionnaire was distributed to ascertain attitudes towards RMS, including obstacles to their use and how these might be overcome.

**Results:**

Monthly medium size plastic specula use decreased during the pilot, resulting in a 22.4% reduction in carbon footprint. Exclusively using RMS for all examinations could reduce carbon emissions by 85.6%. Clinicians had an overall positive attitude towards RMS. Key obstacles to use were poorly stocked rooms and insufficient variety of size.

**Discussion:**

The success of this pilot scheme is being built upon by investing in a greater variety of sizes of RMS for use in SHAC aiming for a 100% reusable system.

## Introduction

Climate change is a direct threat to human health, and mitigation of this damage is essential in healthcare provision.^1,2^ The National Health Service (NHS) is England’s largest public sector organisation and contributes 4% of the national carbon footprint and produces 156,000 tonnes of clinical waste each year.^3^ To recognise this responsibility, in 2019 the NHS committed to net zero direct emissions by 2040.^4^ Life Cycle Analyses (LCAs) conducted in the United States have shown that using stainless steel reusable specula reduces greenhouse gas emissions compared to disposable plastic alternatives.^5^ However, research into the practical introduction of RMS in clinical practice is lacking.

The Sexual Health and Contraception (SHAC) department at University Hospitals Sussex (UHS) in southern England has two main sites for outpatient care, where both clinics have multidisciplinary teams (MDT) working to provide diagnoses and treatments for a broad range of SHAC conditions. Some members of the SHAC MDT are part of a Green Team dedicated to integrating sustainability into clinical practice. This team conducted a survey in 2021 to determine opinions from staff and patients and found that both groups were highly accepting of RMS.^6^

The aim of this study was to review the pilot with a view to expand the use of RMS within the SHAC department. The analysis included: consumption change of plastic speculum during the pilot; the carbon emissions and cost implications; practitioner attitudes towards reusable metal specula including barriers and facilitators of use; and outcomes if RMS became standard of care for every examination.

## Methods

### RMS implementation

The pilot trial of RMS ran between November 2022 to September 2023. In November 2022 50 medium-sized RMS were introduced for use at one clinic site, and in June 2023 another 50 were introduced at the second site, due to staff requests. After use in clinic, each metal speculum and its identifying tag must be put into a paper bag and transported to be sterilised at the Royal Sussex County Hospital (RSCH) Sterile Services Department (SSD), which is in proximity with both clinics (∼1 mile). Examination rooms were provided with both reusable metal and disposable plastic specula to cover the range of sizes required.

Usage data was gathered from the SSD and Procurement. Using the identification tags it was possible to track how many RMS were being used by SHAC each month from November 2022 (the start of the pilot) to September 2023. Purchasing data was gathered for every month from August 2021 to September 2023 across all sizes of plastic speculum. Microsoft Excel was used to format the data and calculate monthly and daily mean usage.

### Carbon estimates

Carbon estimates were calculated on the assumption that the plastic and metal specula in this study had the same initial carbon footprint as those in the Donahue LCA (regardless of size): plastic was 0.88kgCO2e per speculum, metal was 2.48kgCO2e per speculum.^5^ Carbon emissions from sterilisation were assumed to be equivalent to that of a single individually wrapped instrument in Rizan’s study of instrument sterilisation at the RSCH,^7^ which is the study site and uses renewable energy.

### Costs

Plastic specula costs were calculated using data from procurement. RMS initial purchase costs were divided over 20 years (expected lifespan) to allow fair comparison. Cost of repair or replacement of RMS was not accounted for in this calculation. For the potential scenario where all examinations used RMS it was assumed that the department would purchase another 160 specula to cover all sizes. No additional sterilisation costs were incurred by the SHAC department as these were already included in Trust overhead costs.

### Staff follow-up questionnaire

An anonymous healthcare professional (HCP) questionnaire was distributed to all clinical staff at both clinic sites via email during the pilot. Questions included confidence and use of metal specula, enablers and barriers to use, and if there were any unexpected benefits to the metal specula with options of writing comments in free text boxes. Microsoft Excel was used to process quantitative data, and free-text responses were categorised by key words and themes.

## Results

### Carbon estimates and usage data

**Figure 1.**
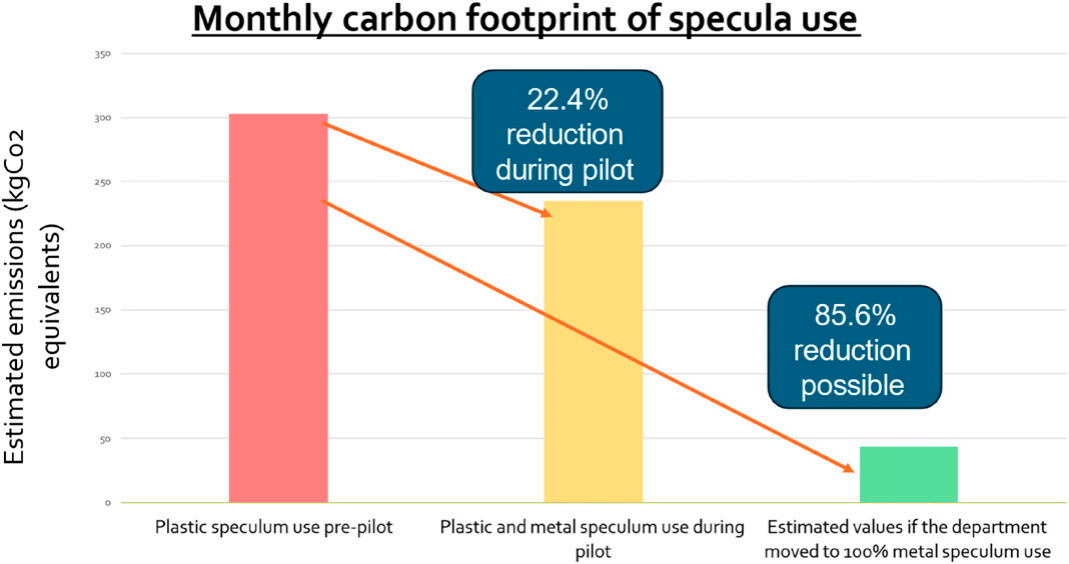
Monthly carbon emissions from vaginal specula before and during the pilot, and potential emissions from an all-reusable system.

Before the pilot, approximately 344 disposable plastic specula were used per month incurring an approximate carbon footprint of 302.02 kgCO2e. During the pilot the average utilisation of plastic specula decreased by 23.8%–262 per month. This reduced the carbon footprint of speculum use by 22.4%–234.77kgCO2e (Figure 1). If the department transitioned to a fully reusable system for vaginal specula, the monthly carbon footprint could be reduced by as much as 85.6% (43.70kgCO2e per month). Over an entire year, the carbon saved would be equivalent to the emissions a small petrol car would produce driving 10,656 miles.

### Costs

The average price of one plastic speculum was £0.59 regardless of size. The cost of disposal of 1 plastic speculum (around £0.05) was based on the national average cost (2019–2020) for high temperature incineration.^8^ RMS had an average purchase price of £20.62. It was calculated that switching to all-reusable specula could save the department £2000 per year while sterilisation costs are absorbed.

**Figure 2.**
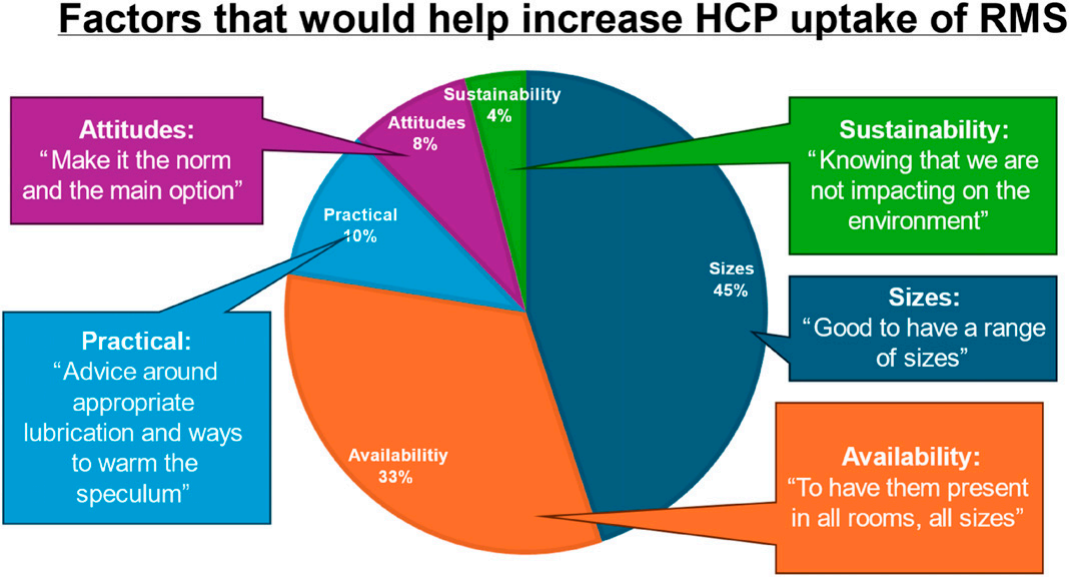
HCP comments on what would help increase their use of RMS in clinic: free-text responses were categorised into 5 themes to highlight salient areas for improvement.

Staff follow-up questionnaire 36 HCPs (56% doctors, 44% nurses) responded to the survey (response rate 92%). HCP experience using the metal specula was broadly positive. 94.4% (*n* = 34) reported they feel confident using a metal speculum. 81% found there was a practical advantage to using RMS, such as feeling sturdier and more robust (with reference to plastic specula snapping in the past), smoother insertion and easier to find the cervix. HCPs made suggestions on how to increase uptake of RMS (Figure 2). Patient experience was not surveyed.

## Discussion

Vaginal specula are an essential part of vaginal examinations in SHAC, and large carbon savings can be made by switching to reusable devices – this was shown to be acceptable to HCPs and easy to implement within the department. This reliable local system of reuse and sterilisation guarantees the availability of RMS into the future.

A key strength of this project was how it highlighted a growing national interest in reusable instruments: the project was presented at the British Association of Sexual Health and HIV national conference in June 2024 and received a high level of interest from colleagues wishing to switch to reusable specula at their Trusts.

This pilot was limited to one department at one hospital, so staff experiences may not be representative of the wider workforce. Sterilisation cost may vary between centres therefore cost savings in this study may not be reproducible. Carbon footprint analysis was estimated for demonstration only and does not accurately represent carbon emissions. Patient experience with RMS was surveyed as part of the initial acceptability questionnaire^6^ as they were already in use for intrauterine contraceptive clinics. This phase of pilot was completed as a medical student research project and therefore had time and scope limitations, so no patient questionnaire was distributed.

The success of this pilot scheme is being built upon by investing in a greater variety of sizes of metal specula for use in SHAC, aiming for 100% RMS use as well as introducing RMS into the HIV clinic. Appropriate measures (such as improved examination lights, opportunities to practice using the metal speculum, improved stocking of speculum drawers and training) will be implemented to improve clinician experiences with using metal specula (Figure 2). Other clinical areas which use vaginal specula may also wish to switch to reusable specula, such as other sexual health, and obstetrics and gynaecology departments. Hospitals with on-site SSD will find it easier to introduce reusable specula as infrastructure for sterilisation is already present but small onsite sterilising machines can be used in community clinics.

## References

[1] World Health Organisation . Climate change and health. https://www.who.int/newsroom/fact-sheets/detail/climate-change-and-health (2023). accessed 16th March 2024.

[2] World Health Organization . Alliance for transformative action on climate and health (ATACH): COP26 health programme. https://www.who.int/initiatives/alliance-for-transformativeaction-on-climate-and-health/cop26-health-programme.

[3] NHS England . NHS clinical waste strategy. https://www.england.nhs.uk/long-read/nhs-clinical-waste-strategy/ (7 March 2023), accessed 16th March 2024.

[4] NHS England . Delivering a ‘net zero’ national health Service. B1728. https://www.england.nhs.uk/greenernhs/wp-content/uploads/sites/51/2022/07/B1728-deliveringa-net-zero-nhs-july-2022.pdf (2022).

[5] Donahue LM Hilton S Bell SG , et al. A comparative carbon footprint analysis of disposable and reusable vaginal specula. Am J Obstet Gynecol 2020; 223(2): 225. 10.1016/j.ajog.2020.02.007 32067971

[6] Ross S Simmons K Clarke A . The attitudes of practitioners and patients towards reusable metal vaginal specula as a sustainable alternative to single use plastic. Sex Transm Infect 2022; 98: A71–A72.

[7] Rizan C Lillywhite R Reed M , et al. Minimising carbon and financial costs of steam sterilisation and packaging of reusable surgical instruments. Br J Surg 2022; 109(2): 200–210. 34849606 10.1093/bjs/znab406 PMC10364739

[8] NHS England . Appendices to the NHS clinical waste strategy. B2159ii. https://www.england.nhs.uk/wp-content/uploads/2023/03/B2159ii-appendices-nhs-clinical-wastestrategy.pdf (2023).

